# My view on your actions: Dynamic changes in viewpoint-dependent auditory ERP attenuation during action observation

**DOI:** 10.3758/s13415-023-01083-7

**Published:** 2023-03-22

**Authors:** Alexander Seidel, Constanze Weber, Marta Ghio, Christian Bellebaum

**Affiliations:** Institute of Experimental Psychology, Department of Biological Psychology, Heinrich Heine University, Universitätstrasse, 1, 40255 Düsseldorf, Germany

**Keywords:** Auditory ERP, Action observation, Viewpoint, Agency

## Abstract

It has been suggested that during action observation, a sensory representation of the observed action is mapped onto one’s own motor system. However, it is largely unexplored what this may imply for the early processing of the action’s sensory consequences, whether the observational viewpoint exerts influence on this and how such a modulatory effect might change over time. We tested whether the event-related potential of auditory effects of actions observed from a first- versus third-person perspective show amplitude reductions compared with externally generated sounds, as revealed for self-generated sounds. Multilevel modeling on trial-level data showed distinct dynamic patterns for the two viewpoints on reductions of the N1, P2, and N2 components. For both viewpoints, an N1 reduction for sounds generated by observed actions versus externally generated sounds was observed. However, only during first-person observation, we found a temporal dynamic within experimental runs (i.e., the N1 reduction only emerged with increasing trial number), indicating time-variant, viewpoint-dependent processes involved in sensorimotor prediction during action observation. For the P2, only a viewpoint-independent reduction was found for sounds elicited by observed actions, which disappeared in the second half of the experiment. The opposite pattern was found in an exploratory analysis concerning the N2, revealing a reduction that increased in the second half of the experiment, and, moreover, a temporal dynamic within experimental runs for the first-person perspective, possibly reflecting an agency-related process. Overall, these results suggested that the processing of auditory outcomes of observed actions is dynamically modulated by the viewpoint over time.

**Supplementary Information:**

The online version contains supplementary material available at 10.3758/s13415-023-01083-7.

The phenomenon of attenuated perceptual intensity and reduced neurophysiological responses for self-produced compared with environmental stimuli (Baess et al., 2011; Sato, 2008) is usually attributed to the inherent predictability of these stimuli. There is disagreement, however, about the contribution and role of motor representations in generating such predictions (Dogge et al., 2019; Korka et al., 2021; Picard & Friston, 2014; Reznik & Mukamel, 2019). While in the framework of *predictive coding*, goal-directed motor actions are assumed to contribute more at a cognitive level (i.e., as an intention or goal) to the prediction of sensory action outcomes (Kilner et al., 2007; Picard & Friston, 2014), motor control theory suggests that motor-based *internal forward models* enable this prediction (Blakemore et al., 2001; Miall & Wolpert, 1996; Wolpert & Flanagan, 2001). According to the latter account, copies of motor commands are sent from the supplementary motor areas to the cerebellum, where sensory consequences of the resulting actions are computed, so that these can be considered in perceptual processing (Blakemore et al., 2001).

In the auditory domain, electroencephalography (EEG) studies consistently reported a reduction of the event-related potential (ERP) component N1 and a later positive component (P2/P3a) for self-produced versus external stimuli (Baess et al., 2011; Horváth, 2015; Schafer & Marcus, 1973). The reduction of the auditory N1 amplitude has been associated with motor-based forward model predictions, as it is less pronounced or absent when motor information is lacking, e.g., for visually cued auditory stimuli (Klaffehn et al., 2019; Lange, 2011; Sowman et al., 2012), or when the forward model is assumed to be compromised, as in cerebellar lesion patients (Knolle et al., 2012, 2013a). The N1 reduction also is less pronounced for involuntary movements (Jack et al., 2021; Timm et al., 2014). On the contrary, in all of these circumstances P2 amplitude reductions have been reported, thus indicating a reliance of P2 modulations on nonmotor predictions. Furthermore, P2 amplitude reductions have been shown to be sensitive to situational context information related to agency, i.e., the sensation of authorship over a stimulus (Kühn et al., 2011; Timm et al., 2016) or the perceived control over stimulus appearance (Seidel et al., 2021).

An aspect that has received less attention so far is the extent to which similar processes that have been proposed to underlie processing of sensations generated by own actions are also shared for sensations caused by observed actions. After the discovery of mirror neurons that discharge both when a goal-directed action is executed and observed (Bonini, 2017; Di Pellegrino et al., 1992; Mukamel et al., 2010; Rizzolatti & Sinigaglia, 2016), it was hypothesized that observed actions also might trigger motor-based forward model predictions, which in turn might modulate sensory processing during action observation, similar to action performance (Wolpert et al., 2003). This hypothesis has been addressed by few electrophysiological studies to date (Ghio et al., 2018, 2021; Poonian et al., 2015). In our previous studies, we found reduced auditory P2 amplitudes for sounds elicited by actions observed on a computer screen (Ghio et al., 2018) and in person (Ghio et al., 2021), whereas N1 amplitudes were reduced only in the former study. One possible explanation for the differential pattern of the N1 may be related to the difference between the studies in stimulus timing. In the study with on-screen observation (Ghio et al., 2018), there was a delay of nearly 200 ms between observed button press onset and tone, whereas in the study with in-person observation (Ghio et al., 2021), there was a delay of approximately 50 ms. In the latter study, the delay may have been too short for action-related information to affect early processing of the action-related sound, as for action observation the motor system is activated later compared with self-action (Sebastiani et al., 2014). Furthermore, study differences in terms of their setting may have contributed to the different result pattern in the sense that motor action animations with standardized, time-controlled visual stimuli (as in the study with on-screen observation, Ghio et al., 2018), in contrast to a naturalistic setting (as in the study with in-person observation Ghio et al., 2021) might have facilitated a motor-based prediction during action observation, possibly in form of an internal forward model, as described above. Similarly as for self-performed action, a reduction of the P2 in both of our previous studies could then be interpreted to reflect a more general predictive mechanism in action observation, which does not necessarily rely on precise motor-related information.

Interestingly, neurophysiological responses by nonhuman primates and humans during action observation also have been shown to be sensitive to the observer’s viewpoint (first-person versus third-person). For instance, it has been shown that a large proportion of single cells in the monkey premotor area F5 shows a selective preference, that is, a stronger discharge, for one specific compared with other tested viewpoints (Caggiano et al., 2011; Maranesi et al., 2017). Moreover, a modulation by viewpoint has been shown for local field potentials in the monkey area F5 (Caggiano et al., 2015). Observing motor actions from a first-person compared with a third-person perspective was associated with a significantly stronger power increase in the low-frequency band (2-10 Hz), which also is found during action execution. In humans, mu-rhythm suppression during action observation, which is regarded to reflect “mirror neuron activity” (Pineda, 2005), has been shown to be stronger during first-person compared with third-person observation of reach-to-grasp actions (Angelini et al., 2018; Drew et al., 2015; Fu & Franz, 2014).

This indicates a specialized processing of actions seen in first-person, supporting the notion that correlated visual and motor/proprioceptive experience, which occurs more frequently with a first-person perspective (e.g., when monitoring own actions for correctness during execution) than with a third-person perspective (e.g., when copying the actions of a dance teacher) is key to their neurophysiological coupling (Heyes, 2001). A stronger association between own actions and actions observed from a first-person compared with a third-person perspective also might facilitate sensory predictions relying on motor information and therefore might have (additionally) contributed to the differential pattern of findings of our previous studies, as the animations used by Ghio et al. (2018) showed another person’s hand performing button presses in a first-person perspective, while participants in Ghio et al. (2021) observed a person sitting next to them, i.e., from a third-person perspective. A systematic testing of the effects of the observer’s viewpoint on sensory predictions, however, is so far missing.

Furthermore, another crucial aspect that has only recently been considered is that motor-based sensory prediction can change within a short period of time, consistently with the adaptive nature of internal models (Miall & Wolpert, 1996). For instance, Kilteni et al. (2019) demonstrated that exposure to a systematic delay (100 ms) between the execution and reception of a self-generated touch led to a decrease of perceptual attenuation for immediately delivered self-initiated touch and an increase of the attenuation for the delayed touch, representing a retuning of the internal (forward) model. As has been pointed out by Dogge et al. (2019), such learning mechanisms may be particularly relevant for predictions concerning environment-related (as opposed to body-related) action outcomes, such as the sounds resulting from button presses in the self-generation paradigm. In one EEG study in the auditory domain, Timm et al. (2016) showed that after exposure to a systematic delay (200 ms) between the action execution and a self-generated sound, amplitude attenuations for self-generated sounds presented immediately and without this delay (versus visually cued external sounds) were reduced, although only for the P2 and not the N1 component. Furthermore, Schneider et al. (2018) showed that mice learn to selectively suppress reafferent auditory cortical responses to auditory sensations that are coupled to their movements via training in an acoustic virtual reality system. Thus, also for observed actions increasing exposure over the course of the experiment could allow for the tuning of an internal model of the observed action and its effect, which would be reflected in dynamic changes in the processing of the action outcome. A possibility to examine such changes over the course of an experiment is to model variables for the time course in multilevel modeling, which is an increasingly popular statistical approach that also has been applied to the analysis of auditory ERPs in recent years (Bolt & Loehr, 2021; Pinheiro et al., 2019; Seidel et al., 2021; for an overview, see Volpert-Esmond et al., 2021). Applied on trial-level data, it allows modeling of trial-to-trial variability and, thus, dynamic changes in the processes underlying ERP components within an experiment. These processes may comprise confounding variables, such as fatigue, as well as variables of interest, such as the above-mentioned learning processes.

The present study addressed the question whether the viewpoint during action observation can affect the processing of auditory consequences of observed motor actions by employing a purely observational version of the standard self-generation paradigm (Horváth, 2015) to test ERP modulations for sounds elicited by actions observed from a first- versus third-person perspective compared with externally generated sounds. Participants watched videos of actors producing sounds by button presses from both viewpoints, while we recorded EEG data and ocular gaze position on the screen. For both viewpoint conditions, sounds were presented around 300 ms after the onset of button press animation, thus enabling a motor prediction available in early auditory processing. To consider potential dynamic changes over time in the processing of auditory consequences of observed motor actions and examine whether the modulatory effect of the viewpoint appears in a time-variant fashion, we analyzed the data with linear mixed-effects models, as an application of multilevel modelling, on trial-level data. By applying this approach, we not only modelled the effects of Sound Type (action- and externally generated sounds) and Viewpoint (first- and third-person) as experimental factors to test viewpoint-dependent auditory ERP attenuation for action-generated versus externally generated sounds during action observation, but we also modelled the temporal structure of the experiment to consider potential learning processes in action observation. More specifically, we included two predictors that model *time* on different levels, that is, a) the predictor Run to account for the division of each experimental condition in two identical experimental runs presented, respectively, in the first and second half of the experiment, and b) the predictor Trial number to model developments with increasing trials within each run for each condition (see *Methods*).

For the N1 component, in line with the hypothesis that the mirror neuron system might be involved in generating action-observation based predictions, at least when the delay between action and its consequence is long enough to enable a motor prediction available in early auditory processing (Ghio et al., 2018), we expect the amplitude to be reduced for observed action- compared to externally generated sounds for both viewpoints. Furthermore, based on evidence for a stronger involvement of the mirror neuron system for a first-person perspective (Angelini et al., 2018; Fu & Franz, 2014), we expected a stronger N1 reduction for sounds resulting from observed actions for the first- than the third-person perspective condition. Effects of the time course that interact with the type of sound are of particular interest, as such interactions could reflect learning processes in action observation. Consistent with the role of learning in predicting environmental action outcomes (Dogge et al., 2019), we expected that the general N1 attenuation for self-generated sounds would become stronger over the course of the experiment. Effects of the time course that, in addition to the type of sound, depend on the viewpoint during observation could indicate a difference in the ease of learning between viewpoints. Because learning a motor-based prediction, as hypothesized for the N1, might be facilitated by a stronger motor involvement as associated with a first-person perspective during action observation, we expected the increase in N1 attenuation over time to be stronger for the first-person than for the third-person perspective.

Regarding the P2 component, previous studies suggested that this component is not sensitive to motor-related, but rather general, context-dependent predictions (Knolle et al., 2013a; Seidel et al., 2021), and thus it is unlikely that differing mirror neuron system activity in the first- versus third-person perspective would affect this component. Because the motor action to be observed cues sound onset for both viewpoints equally and, thus, allows context-dependent predictions, we expected the P2 to be reduced for observed action-generated compared with externally generated sounds irrespective of the viewpoint conditions, but, in contrast to the N1, without a stronger reduction for the first- versus third-person perspective (as this was hypothesized to reflect a stronger involvement of the mirror neuron system in action-based prediction mechanisms not involved here). On the contrary, given that P2 amplitude reductions have been shown to be sensitive to agency ambiguity (Kühn et al., 2011; Timm et al., 2016), we speculated that the first-person perspective may introduce such an ambiguity, as it is similar to looking at one’s own hands. This could result in a diminished P2 reduction compared to the third-person perspective, which emphasizes the self-other distinction, and may thus enable an easier agency judgement. Concerning the effects of the time course, we can hypothesize that the suspected advantage in agency attribution for the third-person perspective might become less strong over the course of the experiment, as any initial agency ambiguity for the first-person perspective might dissolve over time, reflected in an increase in P2 attenuation over time only for the first- but not for the third-person perspective.

## Method

### Participants

Twenty-seven participants (13 females, 14 males, mean age 23.7 years ± 4.4) with normal or corrected-to-normal vision and normal hearing took part in the experiment. The sample size was thus slightly larger compared to our previous studies where we found within-subject modulations of the auditory attenuation effect in groups of 20 participants (Ghio et al., 2018, 2021). Except for one participant, all reported to be right-handed. None of the participants reported a history of neurological disease, mental disorder or current medication affecting the central nervous system. Informed written consent was obtained from each participant before the experiment. Participants received either course credit or money as compensation. This study was approved by the Ethics Committee of the Faculty of Mathematics and Natural Sciences at Heinrich Heine University Düsseldorf, Germany.

### Materials

#### Visual stimuli

**Fig. 1:**
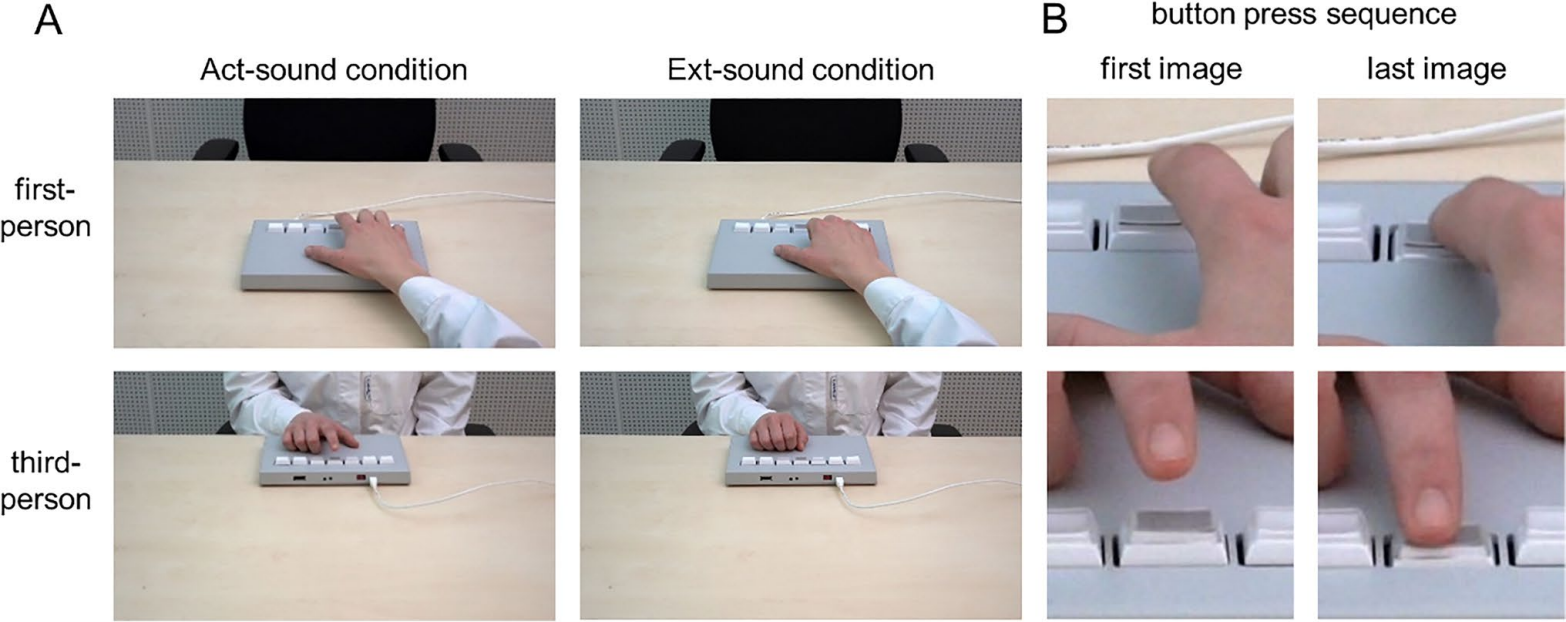
**A**: Images from the first- and third-person perspective showing the female actor holding the right index finger in the starting position for each button press (Act-sound condition) and resting the right hand on the button box (Ext-sound condition). **B:** Close-up of the first and the last image (of the sequence of 10 images) of the Act-sound condition from the first- and third-person perspective, showing the right index finger of the female actor in the starting position and while fully pressing the button. Please note that the same images used for the button press sequence in the Act-sound condition also were used for the Motor-only condition

As outlined in the Introduction, the purpose of the experiment was to study the processing of sounds that were elicited by observed button presses. We therefore recorded videos showing the hand of either a male or female actor pressing a button on a Cedrus RB-740 response pad (www.cedrus.com) with their right index finger. Furthermore, the viewpoint was varied. The button presses were shown either from the first- or from a third-person perspective, so that there were four versions of the video in total. The observed persons wore a white lab coat, which was identical to the one worn by the participants during the EEG recording (see below), and the videos were recorded in the same EEG-chamber in which the EEG data acquisition was conducted. Figure 1A contains example images showing the female actor (for further information on the video recording, including example images of the male actor, see supplementary material S1).

For visual stimulation during the experiment, however, ten consecutive images (resolution 1920 × 1080 px) were extracted from each video and were shown in succession (see below), so that the ERPs could be time-locked to comparable points in time during the observed button press for each version. The first image of each sequence showed the finger above the button, while in the last image the button was fully pressed (Fig. 1B). During the experiment, the images were shown on a 60-Hz monitor, with consecutive images appearing every second frame, so that each image was shown for approximately 33 ms, which led to the impression of a fluid button press motion (see below for further details). In addition to the images for the observed button press, one image was taken per version (female or male in first- or third-person perspective), which showed the hand of the actor on the button box in a closed fist (Fig. 1A). This image was used for the condition in which no button presses were observed (see below).

#### Auditory stimulus

The sound played during the experiment was created with MATLAB R2019a (MathWorks Inc., Natick, MA) and delivered via over-ear headphones (Sennheiser HD 201) with the same duration and pitch for all experimental conditions (1000 Hz, 200-ms duration, 20-ms fade in/out).

### Experimental design

We adapted the block designed self-generation paradigm (Horváth, 2015), which usually involves active button presses by the participants, in order to create an observational variant of it. Although the participants of the present study only engaged in an observational variant of the self-generation paradigm and never performed actions themselves, we will use the labels for the experimental conditions that are usually used in active versions of the paradigm (see previous studies involving observational versions of the self-generation paradigm, Ghio et al., 2018, 2021). The paradigm involved the observational variant of the three standard conditions of the self-generation paradigm (Horváth, 2015). In one condition, the observed actor performed a sequence of button press actions that elicited sounds (Act-sounds). In another condition, externally generated sounds (Ext-sounds) not preceded by an observed button press were played. Finally, in the Motor-only condition, the observed actor performed button presses without producing sounds. The Motor-only condition merely served to control for effects of movement observation on the ERPs (see below for details), and only the ERPs from the motor-corrected Act- and Ext-sound conditions entered the analysis. Importantly, and in accordance with the main purpose of the present study, the viewpoint of the observed action (first- versus third-person) was added as a further factor to the paradigm, yielding a 2 x 2 experimental design, with the factors Sound Type (Act-sounds, Ext-sounds) and Viewpoint (first-person, third-person) as within-subject factors. The different conditions are explained in detail in the following.

#### Act-sound condition

**Fig. 2:**
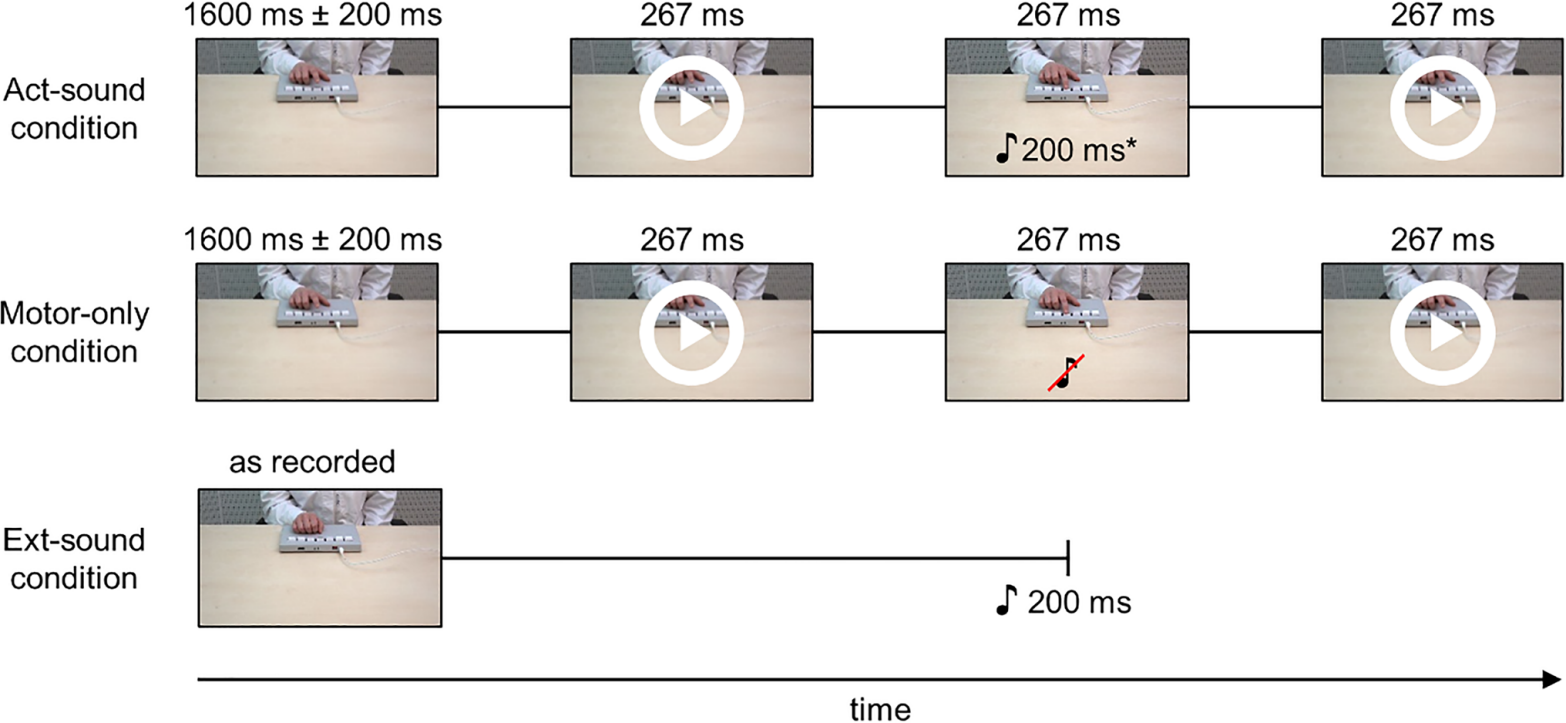
Experimental sequence for the three conditions of the observational variant of the self-generation paradigm, with example images from the female actor from the third-person perspective. The images overlaid with a white “play” sign represent the start of an animation of 8 images at a rate of 33.3 ms. Presentation times are rounded. *Sound onset approximately 30 ms after image onset

Trials of the Act-sound condition started with the presentation of the first image of the button press sequence according to the condition (first- or third-person), showing the finger of a female or male actor (gender-matched to the participant) in the starting position above the button for an average of 1600 ms (± 200 ms random variance). Then, the next nine images in the sequence were shown consecutively to illustrate a button press by the observed person. Eight images were shown for approximately 33 ms each; the last one showed the button fully pressed was presented for ca. 267 ms. Then, the images were shown in reversed order with identical timing to illustrate the button release. The total duration of the observed button press and release was ca. 800 ms. The average interval between observed button presses was 2400 ms (Fig. 2). Importantly, the tone was time-locked to the image showing the button fully pressed, with a delay of approximately 30 ms. It thus appeared ca. 300 ms after the start of the observed button press. Participants were instructed to observe the actions and listen to the sounds attentively. To ensure that participants focused on the button press action, binocular gaze positions were continuously recorded using a dark pupil eye-tracker (see below for details on eye-tracking data acquisition and processing).

#### Ext-sound condition

During the Ext-sound condition, all sounds from the previous Act-sound condition were replayed with the same timing, but importantly they were not preceded by an observed button press. Instead, the image of the resting hand (again gender-matched to the participant) was continuously presented. In this sense, Viewpoint (first- versus third-person) also was varied in the Ext-sound condition, although no button press actions were performed. Participants were instructed to attentively fixate the button and listen to the sounds.

#### Motor-only condition

Because the Motor-only condition is usually employed in the self-generation paradigm to account for electrophysiological responses solely driven by the motor action (Horváth, 2015), we applied an analogous correction procedure for observed button presses as we did in our previous work (Ghio et al., 2018, 2021). The visual stimulation in the Motor-only condition was identical to the visual stimulation in the Act-sound condition, but no sounds were presented. Participants were instructed to attentively observe the button press actions. Binocular gaze positions were continuously recorded in this condition as well to ensure that participants focused on the button press action.

### Experimental procedure

Participants first completed the consent form and a brief demographic questionnaire. For the EEG acquisition session, during which also eye-movement data were recorded, participants were seated in an electrically and acoustically shielded chamber, in front of a 22-inch LCD monitor with a resolution of 1680 × 1050 px. A chin-rest was placed at a preset height for a fixed viewing distance of 62 cm to the screen.

Following the instructions, 11-trial versions of the Act-sound and Ext-sound conditions were presented, once from each viewpoint to familiarize participants with the experimental procedures. This was followed by four experimental runs, each consisting of the three conditions as separated blocks in a fixed order (i.e., Act-sound, Ext-sound, Motor-only). The different runs alternated between showing the observed person from the first- or third-person perspective, with the starting viewpoint counterbalanced between participants. This resulted in two identical runs per Viewpoint, one in each half of the experiment, yielding the two-level predictor Run in the analysis. Before each condition, participants were informed whether they would observe button presses and/or listen to sounds and could take a self-administered break before starting the condition. Fifty-one button presses and/or sounds were presented within each condition in each run, the first of which was disregarded in the analyses, for a total of 100 Act- and 100 Ext-sounds entering analysis for each viewpoint (50 from each run). Stimulus presentation was controlled via Presentation® software (Version 20.3, Neurobehavioral Systems, Inc., Berkeley, CA, www.neurobs.com). To assure that the time delay between sounds and the corresponding EEG sound markers was minimized, a Sound Blaster Audigy Rx (Creative Technology Ltd., Singapore) in “Bit Accurate Playback” mode was used and the Presentation Mixer was set to “exclusive.”.

### Eye movement data acquisition and analysis

For each condition involving button presses, binocular gaze positions were continuously recorded using a dark pupil eye-tracker (iView X RED 500, SensoMotoric Instruments) to ensure that participants focused on the button press action. A 9-point calibration of the eye tracker was conducted before each condition. After calibration accuracy was briefly checked by the experimenter, a focus point for observing the actor’s finger was established for the upcoming condition block. For this purpose, the first image of the button press sequence for the upcoming condition was presented, and participants were instructed to focus their gaze on the finger for one second and press the space bar. The gaze position detected by the eye tracker at this moment was then displayed and confirmed by the experimenter if it was in the general vicinity of the finger. Otherwise, the focus detection or the entire calibration procedure was repeated. This focus point was saved for each condition for the later analysis of the gaze position data. To familiarize participants with this procedure, it also was performed for the two Act-sound condition trainings, but data were not recorded.

Eye movements were recorded at a sampling rate of 500 Hz with the iView X software (Version 2.8). The raw gaze data (extracted with the IDF Converter 3.0.20, SensoMotoric Instruments) was analyzed offline in MATLAB R2019a. After excluding gaze positions that were detected in only one eye, each trial in the Act-sound and Motor-only condition was checked to determine whether the participants focused on the observed button press. For all gaze positions occurring in the 300 ms after the onset of the observed button press (first frame of the second image of the sequence), and thus until the tone was presented, we calculated the distance to the focus point established after the eye tracker calibration for the respective condition in each block. Trials were excluded from further analyses if less than 75% of gaze positions in the 300-ms interval were within 200 px (approximately 5° viewing angle) of the individual focus point. The corresponding trials in the following Ext-sound condition were excluded as well to ensure that the trials that entered analysis were preceded by identical inter-sound-intervals in both conditions. Two participants for whom more than 25% of trials had to be excluded due to this procedure were excluded from further analyses.

### EEG data acquisition and analysis

#### EEG acquisition

EEG was continuously recorded after the start of the first experimental run with Ag/AgCl passive electrodes positioned according to the 10-20 system at a sampling rate of 1000 Hz and referenced to linked mastoids during acquisition. The signal was amplified using a BrainAmp Standard amplifier and recorded via BrainVision Recorder software (Version 1.21.0402, Brain Products GmbH, Germany). Four electrodes were used to measure the electrooculogram (electrodes F9 and F10 for horizontal, Fp2 and a separate electrode below the right eye for vertical eye movements). AFz position was used for the ground electrode. Impedances were kept below 5 kΩ. The other 28 electrodes were placed at the following positions: F7, F3, Fz, F4, F8, FT7, FC3, FCz, FC4, FT8, T7, C3, Cz, C4, T8, CP3, CPz, CP4, P7, P3, Pz, P4, P8, PO7, PO3, POz, PO4, and PO8.

#### Preprocessing

Preprocessing was performed with BrainVision Analyzer software (Version 2.1.2, Brain Products GmbH, Germany) and MATLAB R2019a. After a global direct current de-trend, Butterworth zero-phase filters (low cutoff: 0.3 Hz, 24 dB/oct; high cutoff: 30 Hz, 24 dB/oct), a notch filter (50 Hz), and a semiautomatic independent component analysis (ICA, steps = 512) for the removal of ocular artifacts were applied. The corrected data were segmented into epochs of 700 ms, starting 200 ms before sound onset (a muted sound stimulus was played in the Motor-only condition for this purpose). Segments underwent an automatic artifact rejection (maximal allowed voltage step: 50 μV/ms, maximal allowed difference of values within 100-ms intervals: 100 μV, maximal/minimal allowed amplitude: ± 100 μV, lowest allowed activity within 100-ms intervals: 0.5 μV) and were subsequently baseline corrected using the interval of 200 ms before (muted) sound onset.

Similar to studies in which sounds were actively produced by motor actions (Horváth, 2015), activity evoked by motor observation was removed from the Act-sound segments (similar to Ghio et al., 2018, 2021). The motor correction was applied for each participant, Viewpoint and Run separately. For this purpose, segments of the Motor-only condition were averaged, separately for the first and second run in the experiment, the first- and the third-person perspective and each participant. Then, we subtracted the averaged Motor-only segment (run- and viewpoint-specific) from each individual Act-sound segment (of the corresponding viewpoint and run) to enable analysis based on single-trials (for a visualization of the grand averages of the uncorrected and corrected Act-sounds and Motor-only segments, see supplementary material S3). Visual inspection of these grand averages suggested that the ERPs in the motor-only condition might differ between viewpoints. This was explored in a separate analysis reported in the supplementary material S3. Importantly, since the motor correction was performed separately for the two viewpoints, the motor-corrected Act-sound segments (hereafter referred to as Act-sounds) that entered all further analyses were adjusted for such differences in activity evoked by the motor observation per se and were intended to reflect sound processing only.

**Fig. 3:**
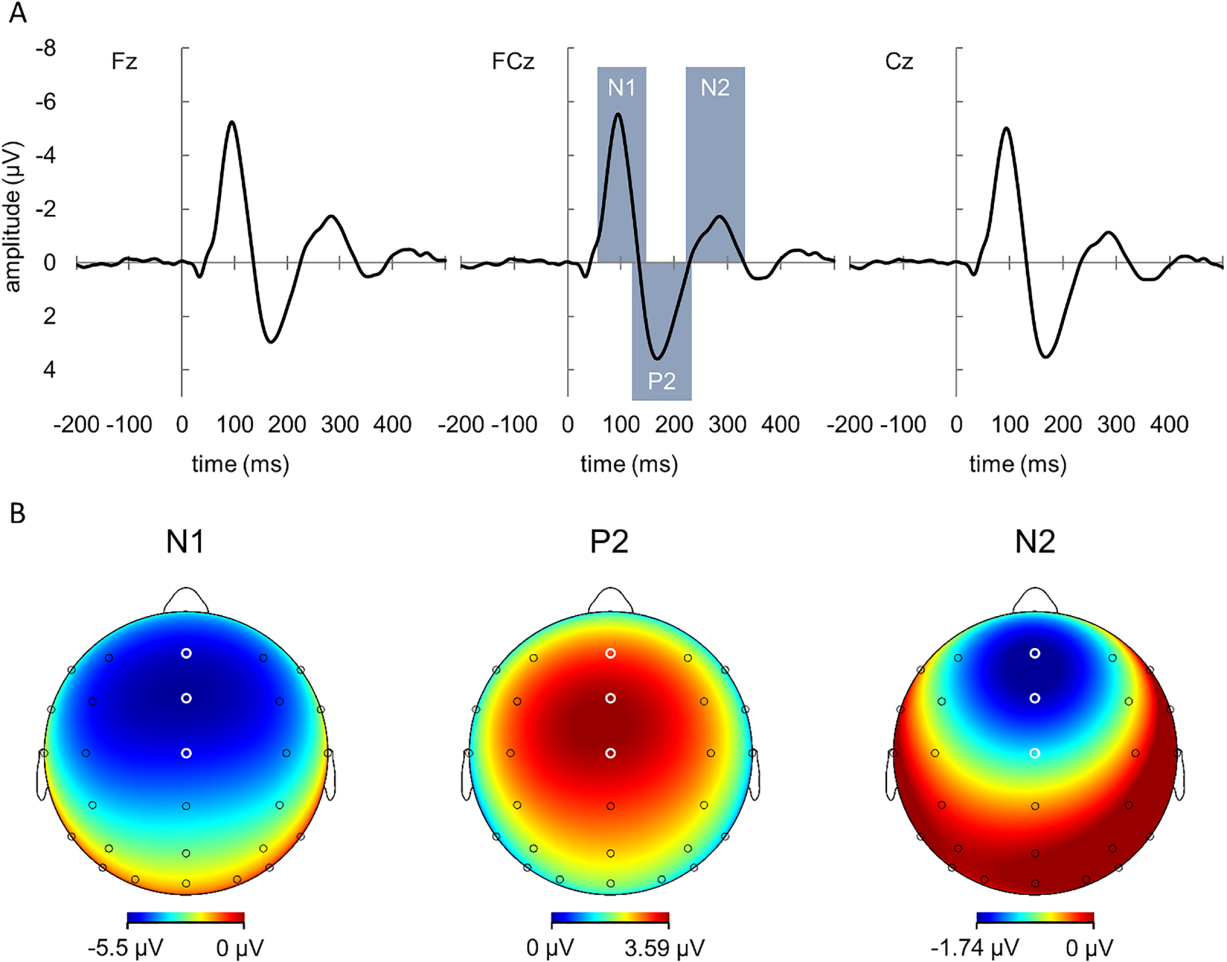
**A:** Overall sound-related grand average ERPs at Fz, FCz, and Cz across Sound Type and Viewpoint conditions, and the time windows (grey rectangles) used for mean amplitude extraction. **B:** Topographical maps showing scalp potentials at the time of the N1, P2, and N2 peaks from the overall grand average ERPs seen in A. White circles indicate positions for the electrodes Fz, FCz, and Cz (from top to bottom)

To determine the ERP components of main interest, we then created an overall grand average across the Act-sound and Ext-sound conditions for both viewpoints (Fig. 3A). Visual inspection of these grand averages suggested that the signal was modulated by the experimental conditions not only in the N1 and P2 time windows, as expected, but also at a negative peak around 300 ms, which also was explored. In accordance with Sugimoto et al. (2021), we will refer to this component as N2. Analysis of the N2 component reported below are exploratory and not based on a priori hypotheses. We determined Fz, FCz, and Cz as the appropriate electrodes for our analyses, based on the topographical maps shown in Fig. 3B for the overall grand average at the peaks of the components of interest (a similar approach was applied in Seidel et al., 2021).

To prepare the dataset to apply linear mixed effects models on trial-level data, data extraction for each component was performed in three steps. First, we localized peaks in the grand average collapsed over all conditions and participants for each electrode. The latencies of these peaks (averaged between electrodes and rounded) were 94 ms (N1), 167 ms (P2), and 284 ms (N2). Second, we determined peaks in the data averaged separately for each Sound Type, Viewpoint, Participant, Electrode, and Run in a time window of 100 ms around the peaks found in the previous step. Since for some subjects, no peaks were found in these time windows, we extended the time window for the P2 (117–230 ms) and N2 (220–334 ms) detection. The N1 detection time window was slightly shortened (50–144 ms) to avoid the selection of a very early separate peak in only one condition for one participant. In a third step, we calculated a mean amplitude value for each trial, averaging the amplitude values from a 40-ms time window centered on the latency of the previously detected peak for this trial's condition and electrode.

For the purpose of detecting outliers in the single-trial mean amplitudes processed for statistical analysis, means and standard deviations were calculated across trials separately for each Sound Type, Viewpoint, Electrode, and Run combination. Single-trial values were removed if they differed more than 2.5 standard deviations from the respective mean. The resulting number of trials per condition (averaged across participants) after preprocessing and outlier-removal can be found in Table 1.

**Table 1:** Average number of trials after preprocessing and outlier-rejection

Dataset	Electrode	Act-sounds first-person M (SD)	Ext-sounds first-person M (SD)	Act-sounds third-person M (SD)	Ext-sounds third-person M (SD)
After preprocessing	All	94.6 (6.5)	94.2 (6.5)	94.4 (7.4)	93.9 (7.8)
After outlier-rejection					
N1	Fz	93.3 (6.8)	93.5 (7.5)	93 (6.8)	92.7 (7.8)
P2	Fz	93.4 (6.3)	93.6 (7.2)	92.7 (6.5)	92.8 (7.4)
N2	Fz	93.6 (6.5)	93.5 (7.3)	93 (6.6)	92.5 (7.7)
N1	FCz	93.3 (6.7)	93.4 (7.4)	93.1 (6.7)	92.9 (8)
P2	FCz	93.6 (6.3)	93.5 (7.1)	92.7 (6.5)	92.9 (7.9)
N2	FCz	93.6 (6.3)	93.4 (7.3)	93.2 (6.5)	92.5 (7.9)
N1	Cz	93.1 (6.6)	93.6 (7.5)	93.3 (6.8)	92.9 (8.2)
P2	Cz	93.6 (6.1)	93.4 (7.2)	92.7 (6.6)	92.7 (7.9)
N2	Cz	93.4 (6.2)	93.3 (7.5)	93 (6.6)	92.4 (7.7)

In preprocessing, trials were removed if the action was not observed (according to eyetracker data), and if they did not pass automatic artifact rejection. The final dataset for each component was determined after individual outlier-rejection, resulting in differing trial numbers per component and electrode. The maximum number of trials per condition was 100

#### Statistical analysis

Single-trial mean amplitude data from the N1, P2, and the N2 component were analyzed separately by applying the same procedure. Specifically, each dataset was fitted, using the restricted maximum likelihood approach, to the same linear mixed effects model, which included the simple coded fixed-effect predictors Sound Type (Ext-sounds [−0.5], Act-sounds [0.5]), and Viewpoint (first-person [−0.5], third-person [0.5]) as the experimental factors of main interest. To model the course of the experiment, as suggested by Volpert-Esmond et al. (2021), two additional predictors were added. As each experimental condition was presented in two identical runs of 50 trials (for a total of 100 trials per condition), one in the first half and one in the second half of the experiment, we included the fixed-effect predictor Run (first [−0.5], second [0.5]) to account for the temporal separation of the two sets of trials. We also added the continuous fixed-effect predictor Trialnumber (1-50) to model developments over the 50 trials in each run. This predictor coded the original temporal position of each trial within each condition and accommodated rejected trials. For example, if trial 8 was rejected, this did not lead to a numbering from 1-49, but from 1-7 followed by 9-50. It is important to note that linear mixed effects models using maximum likelihood estimation techniques are robust to such unbalanced missing observations (Krueger & Tian, 2004). For all participants, the predictor was then centered around the fixed value of 25.5 instead of the actual mean of trial numbers, because this prevented rejected trials from shifting the centering away from the factual middle of the block. While the predictor Run tested the difference between responses in the first versus second half of the experiment and can thus reveal coarse changes in processing, the predictor Trialnumber can provide information on more fine-grained changes within a Run. The model also contained all possible interactions between all the fixed-effect predictors.

Concerning the random effects, to determine the maximal random-effect structure that still allows the model to converge, we started with random intercepts for participants and random slopes for the predictors Sound Type and Viewpoint and their interaction over participants, and random intercepts for electrodes. The only model that converged when adding random slopes for Run and Trialnumber over participants included random slopes for Run over participants, but no interactions with the other predictors. The final model can therefore be described as:

$${\displaystyle \begin{array}{c}\textrm{Mean}\ \textrm{Amplitude}\sim \textrm{Sound}\ \textrm{Type}\ast \textrm{Viewpoint}\ast \textrm{Run}\ast \textrm{Trialnumber}\\ {}+\left(1+\textrm{Sound}\ \textrm{Type}\ast \textrm{Viewpoint}+\textrm{Run}\ \left|\ \textrm{Participant}\right.\right)+\left(1\ \left|\ \textrm{Electrode}\right.\right)\end{array}}$$

Statistical analyses were conducted in R (Version 4.0.3) using the lme4 package (Version 1.1-26). To test for significant effects, *p* values were calculated with the lmerTest package (Version 3.1-3) with Satterthwaite approximated degrees of freedom. In case of significant interactions, we examined them by performing simple effects analyses. For two-way interactions involving categorical fixed effects (e.g., Sound Type, Viewpoint), we calculated two models, in which one of the two predictors involved in the interaction was dummy-coded (0, 1). One model used the first level of the predictor as the reference level, and the other used the second level. For both models, we then tested the main effect of the second predictor involved in the interaction. For two-way interactions involving the continuous predictor Trialnumber (for which dummy-coding is not possible), when resolving by Trialnumber, we centered this predictor around early trials for one model, and around late trials for the other. The centering values of 13 and 38 were determined by adding/subtracting 12.5 (25% of the possible 50 trials per run) from the centering of 25.5, which was used in the main analyses, yielding values that represented the 25th and 75th percentile of the Trialnumber value. For three-way interactions, we subsequently examined the relevant two-way interaction at each level of the first predictor. In the simple effects analyses, the same random effects were specified as in the main analysis. An α level of 0.05 was considered statistically significant. Interactions are only reported when they involve the predictor Sound Type. Analysis code including output (with parameter estimates of all fixed effects for the three analyzed components) can be found at 10.17605/OSF.IO/FGRB3.

## Results

**Fig. 4:**
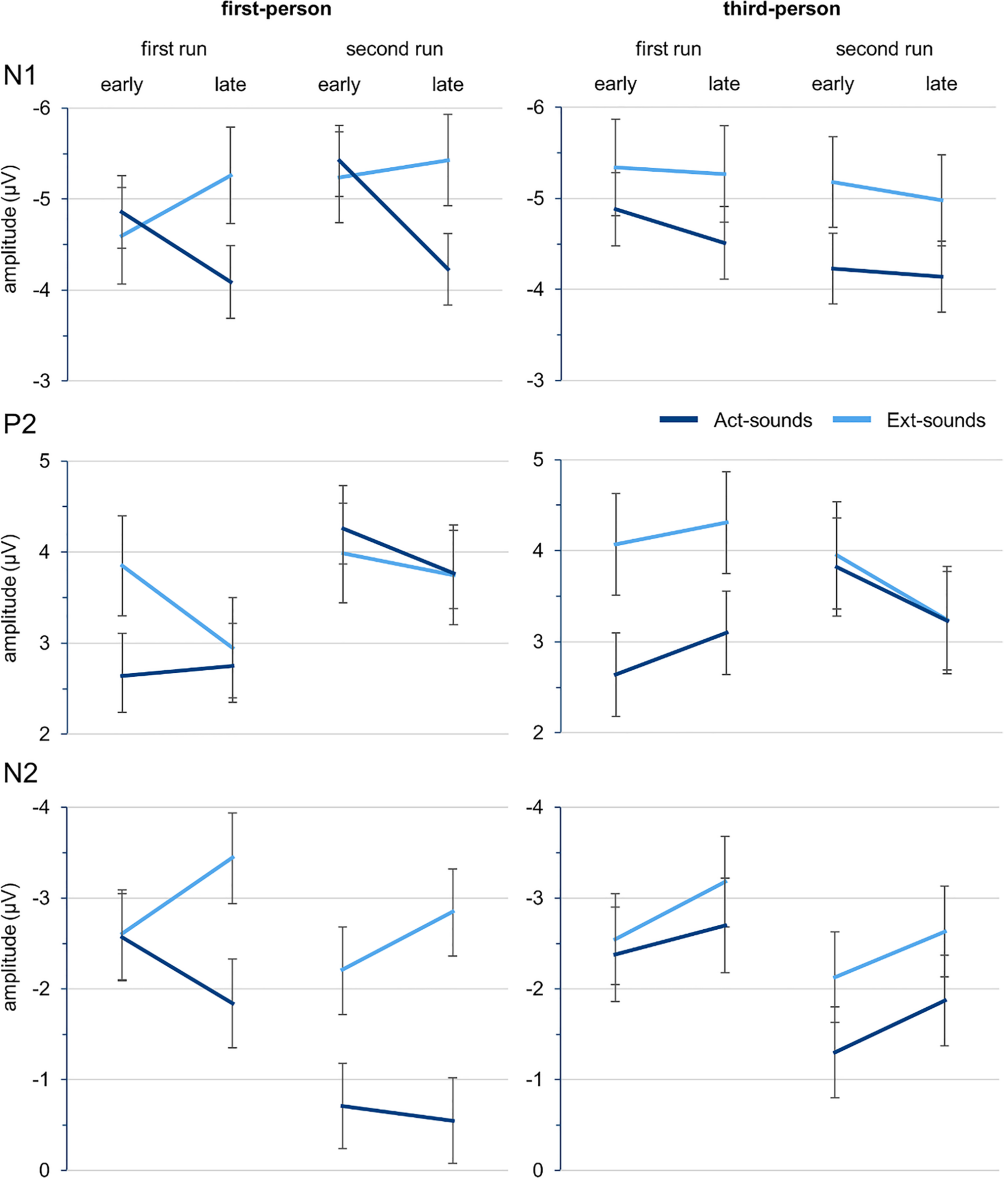
Line plots of the marginal estimated means for the linear mixed effects models. Each run consists of 50 trials, the “early” and “late” trials displayed are trial 13 (25.5 - 12.5) and trial 38 (25.5 + 12.5) of the runs, corresponding to the simple effects analyses. Error bars represent one standard error

For visualization purposes, Figure S3 in the supplementary material shows grand averages in each of the two runs for the Act- and Ext-sound conditions in the first- and third-person perspective. To visualize potential effects of the predictor Trialnumber, grand averages were calculated separately for two bins of trials for each run (trials 1-25 and 26-50, respectively). Figure 4 provides line plots of the marginal estimated means for both types of sounds derived from the linear mixed effects models separately for early and late trials (corresponding to the values tested in follow-up simple effects analyses) in both runs and each viewpoint for the three ERP components that were analyzed.

### N1 component

The model fit for the N1 amplitudes yielded a significant main effect of Sound Type, *F*(1, 25.88) = 8.54, *p* = 0.007, *b* = 0.61, with less negative mean amplitudes for Act-sounds than for Ext-sounds. The main effect of Viewpoint, *F*(1, 25.20) = 0.11, *p* = 0.745, and the Sound Type by Viewpoint interaction did not reach significance, *F*(1, 25.86) = 0.36, *p* = 0.556.

We found a significant interaction of Sound Type by Trialnumber, *F*(1, 30046.18) = 20.58, *p* < 0.001, as well as a three-way interaction of Sound Type by Viewpoint by Trialnumber, *F*(1, 30045.19) = 15.41, *p* < 0.001 (Fig. 4). Simple effects analyses to solve the three-way interaction revealed that the interaction of Sound Type by Trialnumber is only significant for the first-person, *t*(30041.49) = 5.98, *p* < 0.001, but not the third-person perspective, *t*(300048.45) = 0.43, *p* = 0.666. Further simple effects analyses showed that for the first-person perspective, amplitudes for Act-sounds were significantly reduced compared to Ext-sounds in late trials across runs, *t*(33.36) = 3.42, *p* = 0.002, *b* = 1.18, but not in the early trials, *t*(32.65) = −0.65, *p* = 0.519. For the third-person perspective, this reduction for Act-sounds amplitudes were found for early, *t*(34.74) = 2.21, *p* = 0.034, *b* = 0.70, as well as late trials, *t*(34.82) = 2.53, *p* = 0.016, *b* = 0.80. An alternative resolution showed that during first-person observation, amplitudes became significantly less negative with increasing Trialnumber for Act-sounds, *t*(30045.76) = 5.91, *p* < 0.001, *b* = 0.04 (a reduction of 1.96 μV over 50 trials), whereas Ext-sounds amplitudes became significantly more negative, *t*(30045.07) = −2.55, *p* = 0.011, *b* = −0.02 (an increase of 0.85 μV over 50 Trials). During third-person observation, no significant effect of Trialnumber was found, both *p* > 0.157. No other interaction with Sound Type reached significance, all *p*s > 0.363.

### P2 component

Fitting the P2 amplitudes to the model revealed no significant main effects of Sound Type, *F*(1, 26.22) = 3.23, *p* = 0.084, or Viewpoint, *F*(1, 26.00) = 0.04, *p* = 0.846, or Sound Type by Viewpoint interaction, *F*(1, 25.98) = 1.11, *p* = 0.302.

We found a significant Sound Type by Run interaction, *F*(1, 30066.87) = 25.58, *p* < 0.001 (Fig. 4). Follow up simple effects analyses revealed a significant reduction of amplitudes for Act- versus Ext-sounds in the first run, *t*(34.35) = −3.49, *p* = 0.001, *b* = −1.01, but not in the second run, *t*(34.49) = −0.13, *p* = 0.901. All other interactions with Sound Type did not reach significance, all *p*s > 0.105.

### Exploratory analysis: N2 component

The model fit for mean amplitudes from the late N2 time window revealed a significant main effect of Sound Type, *F*(1, 26.05) = 18.55, *p* < 0.001, *b* = 0.96, reflecting less negative amplitudes for Act- compared to Ext-sounds. The main effect of Viewpoint, *F*(1, 25.83) = 0.75, *p* = .394, and the Sound Type by Viewpoint interaction, *F*(1, 26.16) = 2.64, *p* < .116, were not significant.

We found a significant Sound Type by Run interaction, *F*(1, 30055.38) = 12.71, *p* < 0.001. Subsequent simple effects analyses showed significantly smaller amplitudes for Act- compared to Ext-sounds, both for the first run, *t*(39.57) = 2.32, *p* = 0.025, and the second run, *t*(39.90) = 5.42, *p* < 0.001, but parameter estimates revealed that the amplitude difference for the second run (*b* = 1.35) was larger than for the first run (*b* = 0.58).

There were significant interactions between Sound Type and Trialnumber, *F*(1, 30038.60) = 12.27, *p* < 0.001, and between Sound Type, Viewpoint, and Trialnumber, *F*(1, 30036.63) = 8.00, *p* = 0.005. Simple effects analyses to solve the three-way interaction showed that the Sound Type by Trialnumber interaction was only significant for the first-person, *t*(30031.68) = 4.48, *p* < 0.001, but not for the third-person perspective, *t*(30040.93) = 0.48, *p* = 0.634. Subsequent simple effects analyses in first-person perspective revealed significantly reduced Act- compared with Ext-sound amplitudes in early, *t*(33.60) = 2.03, *p* = 0.050, *b* = 0.76, and late trials, *t*(34.37) = 5.15, *p* < 0.001, *b* = 1.95, but with higher parameter estimates for late trials. The analysis for third-person perspective showed no significant Sound Type effect in early trials, *t*(36.29) = 1.48, *p* = 0.148, or in late trials, *t*(36.13) = 1.86, *p* = 0.071. No other interaction with Sound Type reached significance, all *p*s > 0.116.

## Discussion

The present study investigated whether the viewpoint during action observation affects the sensory processing of auditory effects elicited by observed motor actions and whether this hypothesized modulatory effect dynamically changes over time. ERP components associated with auditory processing were compared between sounds generated by actions observed from a first-person and third-person perspective and externally generated sounds during the first versus second run of the experiment. The fine-grained temporal dynamics within each run of the experiment also were examined by modelling the temporal position of each individual trial in each condition. By using multilevel modeling on trial-level data (Volpert-Esmond et al., 2018), we found distinct dynamic patterns of amplitude reductions of the N1, P2, and N2 components for the two viewpoints over the course of the experimental session. While a significant reduction of the N1 component in response to sounds generated by actions observed from a third-person perspective was observed over the entire course of the experiment, a significant N1 reduction when observing from a first-person perspective only emerged later in each experimental run, but was not present in the beginning, i.e., it developed with increasing number of trials. For the P2, we observed a viewpoint-independent pattern over the course of the experiment, i.e., a general P2 reduction for sounds elicited by observed actions in the first but not in the second run, regardless of viewpoint. Our exploratory analyses for the N2 revealed distinct effects between and within runs, with only the latter showing a viewpoint-dependent pattern. A stronger reduction in N2 amplitude was found for the second compared to the first run regardless of viewpoint. While the reduction in response to sounds elicited by actions observed from the first-person perspective increased with increasing number of trials, no such temporal dynamic was found for the third-person perspective.

We hereby partially replicate our previous findings that auditory outcomes of observed actions are processed differently compared with externally generated sounds (Ghio et al., 2018, 2021). Furthermore, we show for the first time that this pattern differs dynamically depending on the viewpoint and time course during action observation, namely that the modulatory effect of viewpoint on auditory ERP attenuation seems to appear in a time-variant fashion.

### N1 component

We expected an overall N1 reduction for sounds produced by observed actions compared with externally generated sounds. Our analyses indeed revealed an overall N1 reduction for sounds following observed actions, in line with our hypothesis and with a previous study from our lab with similar relative timing of observed action and outcome (Ghio et al., 2018). An N1 reduction for sounds caused by motor actions has been interpreted to reflect forward model predictions, which in case of one’s own actions are likely available even before motion onset due to efference copy relayed motor information (Crapse & Sommer, 2008; Reznik et al., 2018). Assuming that, based on the neural substrates of mirror neurons (Bonini, 2017; Di Pellegrino et al., 1992; Mukamel et al., 2010; Rizzolatti & Sinigaglia, 2016), forward model predictions also could be employed during action observation (Wolpert et al., 2003). This also can be an interpretation for an N1 reduction for sounds caused by observed actions.

We also hypothesized that the N1 reduction might be larger during first- versus third-person observation since it has been shown that action observation from a first-person versus third-person perspective evokes stronger mu-rhythm suppression (Angelini et al., 2018), which is regarded to reflect mirror neuron system activity (Pineda, 2005). This hypothesis could not be confirmed, because we did not find a significant interaction between the predictors Sound Type and Viewpoint. With respect to the temporal dynamics, we expected that the N1 reduction for Act-sounds would become more pronounced over the course of the experiment and that this effect would be stronger for the first-person perspective. These hypotheses could only partially be confirmed. We did see an interaction between Sound Type and Trialnumber, hinting at a stronger N1 reduction for Act-sounds in later trials, and this effect was indeed further modulated by Viewpoint. However, during first-person observation, there was no significant N1 reduction at the start of each run, but it developed only toward later trials. This pattern was driven mostly by significantly decreasing Act-sound amplitudes over the course of the experiment but also was amplified by a (relatively smaller) amplitude enhancement for Ext-sounds. For the third-person perspective, in turn, we observed a stable reduction over the entire course of the runs. This pattern of results suggests that, opposite to our hypothesis, the third-person perspective during action-observation facilitates the mechanism underlying the N1 reduction, whereas for the first-person perspective this only develops with time (with increasing Trialnumber). Although an exploratory analysis revealed effects of Viewpoint on the motor-only ERPs before the N1 time window (see supplementary material S2), it seems unlikely that the pattern of results was caused (partially) by differential visual stimulation between viewpoints per se, as we specifically used these viewpoint-specific motor-only control conditions to correct the ERPs of Act-sounds for possible differential activity evoked by movement observation alone (see *Methods*). In the following, we propose three speculative interpretations for our N1 findings.

First, the temporal dynamic for the first-person observation might reflect an increasing precision in the prediction of the observed action-generated sound over the course of the experimental session enabled by ongoing observational motor learning. Observational motor learning can lead to action sequence-specific neural representations in frontoparietal cortex and enhanced performance in action execution similar to physical practice even without an explicit intention to learn (Apšvalka et al., 2018). Because the motor action to be observed in the present study is a simple action which is frequently executed in everyday life (e.g., when typing on a keyboard), a neural representation for the action itself was likely readily available. However, the sensorimotor association between the observed action and its sensory effect was novel and had to be acquired to form a motor-based prediction of the sound. Our finding of a temporal dynamic in the N1 reduction might therefore reflect the formation of this sensorimotor association with ongoing observational practice similarly as shown for physical practice (Burgess et al., 2019). A speculative interpretation for the finding of this temporal dynamic only for first-person observation is that learning from other’s actions (e.g., from a dance instructor) typically involves a third-person perspective in real-life scenarios. This therefore might have enabled participants to predict sensory consequences of actions observed from the third-person perspective after very few trials.

A second interpretation can be that the developing N1 reduction during first-person observation reflects a higher demand on the transformation of the sensory information of the observed action into one’s own motor and visceromotor representation of the action in question (Fu & Franz, 2014). This transformation—also termed as mirror mechanism (Rizzolatti & Sinigaglia, 2016)—might be impeded by a first-person perspective that usually occurs almost exclusively with the observation of own actions and is therefore unfamiliar during the observation of other’s action. The increasing familiarity with the first-person perspective over the course of the experiment may then have facilitated the transformation of the visual input of the observed action into one’s own motor and visceromotor representation of the action, tuning an internal forward model (Kilteni et al., 2019) to predict the action’s sensory consequence and leading to a stronger N1 reduction.

Along similar lines, and as a third interpretation, the temporal dynamic of the N1 reduction during first-person observation could be explained by an initial failure of sensorimotor integration of the visual, proprioceptive and motor signals within an underlying internal forward model (Wolpert et al., 1995). The visual input for the first-person perspective corresponds to the viewpoint one has on the own hand during own actions. The sensorimotor integration of such a visual signal contradicts proprioceptive and (the lack of actual) motor information and might initially result in an ambiguity in the resulting sensory prediction, and thus, a lack of N1 reduction. Increasing familiarity with this combination of converging signals and an accompanying reliability-based reweighting of the signals in their integration (Boyle et al., 2017), might have enabled increasingly accurate prediction by a fine-tuned internal forward model (with the help of inverse models).

Considering that the involvement of any kind of motor-based prediction mechanism has been questioned by studies reporting N1 reductions simply as a result of temporal predictability (Dogge et al., 2019; Kaiser & Schütz-Bosbach, 2018; Sowman et al., 2012), our results appear to provide evidence to the contrary, at least for action observation. The actor-produced sounds we presented were identical in their temporal predictability and should thus have resulted in comparable N1 reductions if unspecific prediction mechanisms had been at work. Instead, the observation of human actions likely provokes specialized processing and results in predictive mechanisms beyond merely neutral visual stimuli (Klaffehn et al., 2019).

### P2 component

For the P2, we also expected a general amplitude reduction for sounds produced by observed actions compared to external sounds since the sound-preceding actions, regardless of viewpoint, allow context-dependent predictions (Knolle et al., 2013a; Seidel et al., 2021). This hypothesis was only partially confirmed. While we did not find a main effect of Sound Type, we observed the hypothesized effect, regardless of viewpoint, for the first experimental run, but the effect disappeared in the second. Our hypothesis that the P2 reduction is generally diminished in the first-person perspective, but increases over the course of the experiment in contrast to the third-person perspective could clearly not be confirmed. While the described interaction pattern between Sound Type and Run suggests a temporal dynamic of the P2 reduction, the effect was not further modulated by Viewpoint, because no interactions involving Sound Type, Viewpoint and any of the two temporal predictors reached significance. Furthermore, the identified viewpoint-independent temporal dynamic appeared in the opposite direction compared to what was expected, i.e., the reduction disappeared instead of becoming stronger over the course of the experiment.

A P2 reduction was consistently reported in our previous studies on auditory consequences of observed actions (Ghio et al., 2018, 2021; van Laarhoven et al., 2021), and it has been associated with the perception of agency in studies examining self-produced sounds (Kühn et al., 2011;Seidel et al., 2021 ; Timm et al., 2016). According to Synofzik et al. (2008), agency can be conceptually split into a feeling of agency, possibly reflected in the N1, and a more conscious judgement of agency, which has been associated with the P2 (Seidel et al., 2021; Timm et al., 2016). At the same time, the P2 has been shown to be attenuated for visually cued sounds (Sowman et al., 2012; but see Harrison et al., 2021) and might therefore at least partly reflect the high temporal predictability that accompanies sounds caused by motor actions, observed and self-performed.

However, these explanations cannot account for the unexpected disappearance of the P2 reduction over time, which we observe in the current study, because both aspects, i.e., agency of the observed action and temporal predictability of its effect, do not change over time. A post-hoc explanation is that a possibly mediating factor that might explain the disappearance of the P2 reduction can be decreasing attention, which is drawn to the visual stimuli over time and might consequently diminish temporal predictability—for both viewpoints equally—and with it the reduction of P2 amplitudes (Sowman et al., 2012). Similarly, relatively heightened selective attention to the consequences of the observed actions (i.e., Act-sounds) in the first run, associated with an ERP termed *processing negativity* (PN) (Näätänen et al., 1978), might have only brought about the P2 reduction in the first place, which then disappeared as selective attention decreased over the course of the experiment. However, this negative shift should have overlapped the potentials in the N1 and N2 time range (Näätänen et al., 1978), which would have manifested as enhanced (i.e. more negative) N1 and N2 amplitudes for Act-sounds and thus less reduced amplitudes compared with Ext-sounds in the first versus second run of the experiment. While we did not observe such an interaction for the N1, we did see an interaction between Sound Type and Run for the N2 that could reflect enhanced Act-sound amplitudes, i.e., a weaker reduction for Act- versus Ext-sounds in the first compared with the second Run.

Another post-hoc explanation for the disappearance of the P2 reduction could be that the processes underlying the P2 reduction in the first experimental run shift in time, i.e., occur earlier or later relative to sound onset, in the second compared with the first run of the experiment. Again, the N2 thereby seems more suitable than the N1, due to the complementary time course of the Sound Type effect compared with the results concerning the P2. Thus, the process underlying the P2 reduction in the first run of the experiment also may have shifted to the N2 time window in the second run (see N2 component section below), rather than both being overlapped by a PN as proposed above. As temporal developments in the reduction of P2 amplitudes in studies examining the processing of self-generated sounds has to our knowledge not yet been analyzed, this might be an interesting avenue for future studies to further characterize similarities and differences during action observation and performance.

### N2 component

The N2 is a negative deflection between 200 and 350 ms that has been associated with the detection of deviants in auditory oddball paradigms (Folstein & Van Petten, 2008). Our exploratory analysis of this component showed an overall reduction for Act- compared with Ext-sounds that was stronger in the second run and a difference in the temporal dynamic within the runs, also shown in Fig. 4. Across both runs, the N2 amplitude reduction for Act-sounds increased over time for first-person, but not for third-person observation.

Using a button-press paradigm similar to the action observation version used here, an enhancement of the N2 was observed for self-generated deviants (in terms of pitch) compared with externally generated deviants, which was interpreted to reflect an increased salience of the deviant when specific (i.e., forward model-based) predictions are at work (Knolle et al., 2013b). Similarly, infrequently delaying the onset of self-generated sounds by 250 ms (compared with a 0-, 50-, and 100-ms delay) elicited a significantly larger N2 compared with for intersound intervals controlled externally generated sounds (Pinheiro et al., 2019). In contrast, a reduction of the N2 for sounds in a sequence of tones was recently observed when participants performed continuous actions to modulate the sequence (in terms of pitch and speed) compared with passively listening to the same sequence afterwards (Sugimoto et al., 2021). Taken together, these studies suggest that the N2 reflects the classification of sound features (Ritter et al., 1979), such as the exact temporal occurrence. Enhanced N2 amplitudes thereby appear to reflect the cognitive detection of unpredicted stimulus properties (Näätänen et al., 1982; Ritter et al., 1992). This can be an interpretation for the current unexpected finding of a stronger reduction of the N2 amplitude for sounds generated by observed actions in the second compared with the first run compared with relatively enhanced N2 amplitudes for externally generated sounds that remain less predictable in their precise temporal occurrence. As outlined above, since we observed a complementary interaction between Sound Type and Run for the P2, this also might have been brought about by a negative shift (i.e., a PN), associated with selective attention, spanning the time range of P2 and N2.

On the other hand, the increasing reduction for the N2 from the first to the second experimental run was complemented by a viewpoint-dependent increase with increasing trials within each run that was not observed for the P2. It therefore seems unlikely that an overlapping PN can fully account for the observed effects of the N2. Specifically, the temporal dynamic within runs was only found for first-person observation, even though sounds in both conditions were equally cued and predictable, suggesting that the more pronounced N2 reduction during first-person observation reflects an additional influence. Interestingly, this increase, found only for the first-person perspective, strongly resembles the pattern we hypothesized a priori for the P2, based on a dissolvement of agency ambiguity for the first-person perspective. Thus, the pattern found for the N2 might also reflect a process, hypothesized for the P2, related to a form of (self-) agency, which might exist due to constant self-observation from this viewpoint, possibly also amplified in the second run by a temporal shift from the P2 to the N2 time range (see above). Nevertheless, a clear interpretation of our unexpected result seems premature, especially since findings on relationships between amplitude reductions and (self-) agency have so far only been reported for self-generated and not for observed action effects and therefore need to be clarified in future studies. Notwithstanding, the current observations can hopefully contribute to a better understanding of N2 variations and their temporal development that might be identified in subsequent studies.

## Conclusions

Using multilevel modeling on trial-level data, this study shows that the processing of auditory action outcomes during action observation is modulated by the viewpoint in a time-variant fashion. As for self-generated sounds, a reduction of N1 amplitudes for sounds caused by observed actions compared with externally generated sounds was found, emerging for both viewpoints. This indicates that similar prediction mechanisms contribute to early auditory processing of sounds following self-performed and observed actions. However, a temporal dynamic of the N1 reduction for the first-person, but not third-person, perspective (i.e., it only emerged with increasing number of trials and was amplified by a relatively weaker N1 increase for externally generated sounds) suggests that viewpoint-dependent mechanisms might be involved in sensorimotor predictions during action observation. A P2 reduction, as commonly found for self-generated sounds, was found regardless of viewpoint for the first but not the second experimental run. Contrary to the P2 finding, an exploratory analysis of the ensuing N2 component revealed a reduction for sounds caused by observed actions compared with externally generated sounds that increased from the first to the second experimental run, as well as a viewpoint-dependent pattern for this increase within runs. Considered together, we speculated that this might reflect a temporal shift of an agency-related process, which affects the first-person perspective more strongly than the third-person perspective, from the P2 to the N2 range over time. Applying trial-level analyses in future studies on the processing of self-generated sounds can help to elucidate whether the temporal dynamics identified here are specific for action observation or are also seen with self-performed actions.

## Supplementary Information

ESM 1 (DOCX 2261 kb)
