# Supplementary material for: My view on your actions: Dynamic changes in viewpoint-dependent auditory ERP attenuation during action observation
Source: Cogn Affect Behav Neurosci. 2023 Mar 22;23(4):1175–91. doi: 10.3758/s13415-023-01083-7 (PMC10400693; doi:10.3758/s13415-023-01083-7)
Supplement: Supplementary file 1 — (DOCX 2261 kb) [file 13415_2023_1083_MOESM1_ESM.docx]

Supplementary online material to:

**My view on your actions: Dynamic changes in viewpoint-dependent auditory ERP attenuation during action observation**

Alexander Seidel^a1^, Constanze Weber^a*1^, Marta Ghio^a^, Christian Bellebaum^a^

^a^Institute of Experimental Psychology, Department of Biological Psychology, Heinrich Heine University, Düsseldorf, Germany

^*^*Corresponding author*: Constanze Weber, Heinrich Heine University, Institute of Experimental Psychology, Department of Biological Psychology, Universitätstrasse, 1, 40255, Düsseldorf, Germany.

Phone: +49 211 81-11594

Email: Constanze.Weber@hhu.de

^1^ co-first authors.

**S1. Creation of visual stimuli**

Four videos (1920 x 1080 px) of button presses were recorded, showing either a male or female actor in a white lab coat (identical to those worn by participants) pressing a button on a Cedrus RB-740 response pad (www.cedrus.com) with their right index finger, once from the first- and third-person perspective each. One button press from each of the four videos was selected that could be extracted as a sequence of ten frames. Each set started with the finger in the starting position, held above the button, and ended with the button fully pressed, as shown with the male actor in Supplementary Figure 1. All frames were converted to images using the media player classic (Version 1.7.13, www.mpc-hc.org). Additionally, four videos were recorded showing the actors resting their hand in a closed fist on the button box in the same position as in the previous videos. One frame from each video was selected and extracted as an image of the resting state using the same procedure as before. All images were presented scaled to the display width during the experiment.
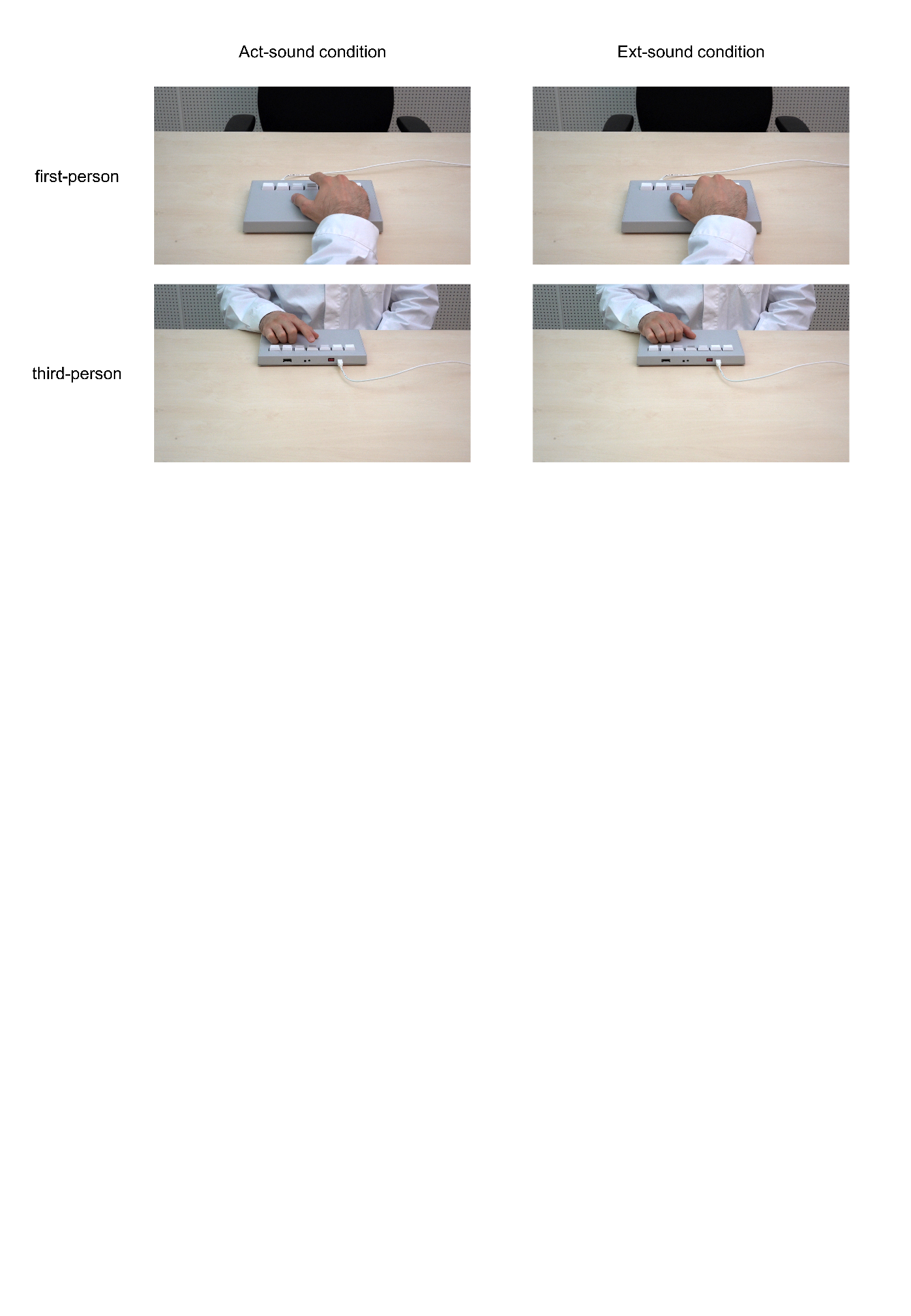


*Supplementary Figure 1.* Images from the first- and third-person perspective showing the male actor holding the right index finger in the starting position for each button press (Act-sound condition) and resting the right hand on the button box (Ext-sound condition).

**S2. Analysis of effects of viewpoint and run on the motor-only condition**


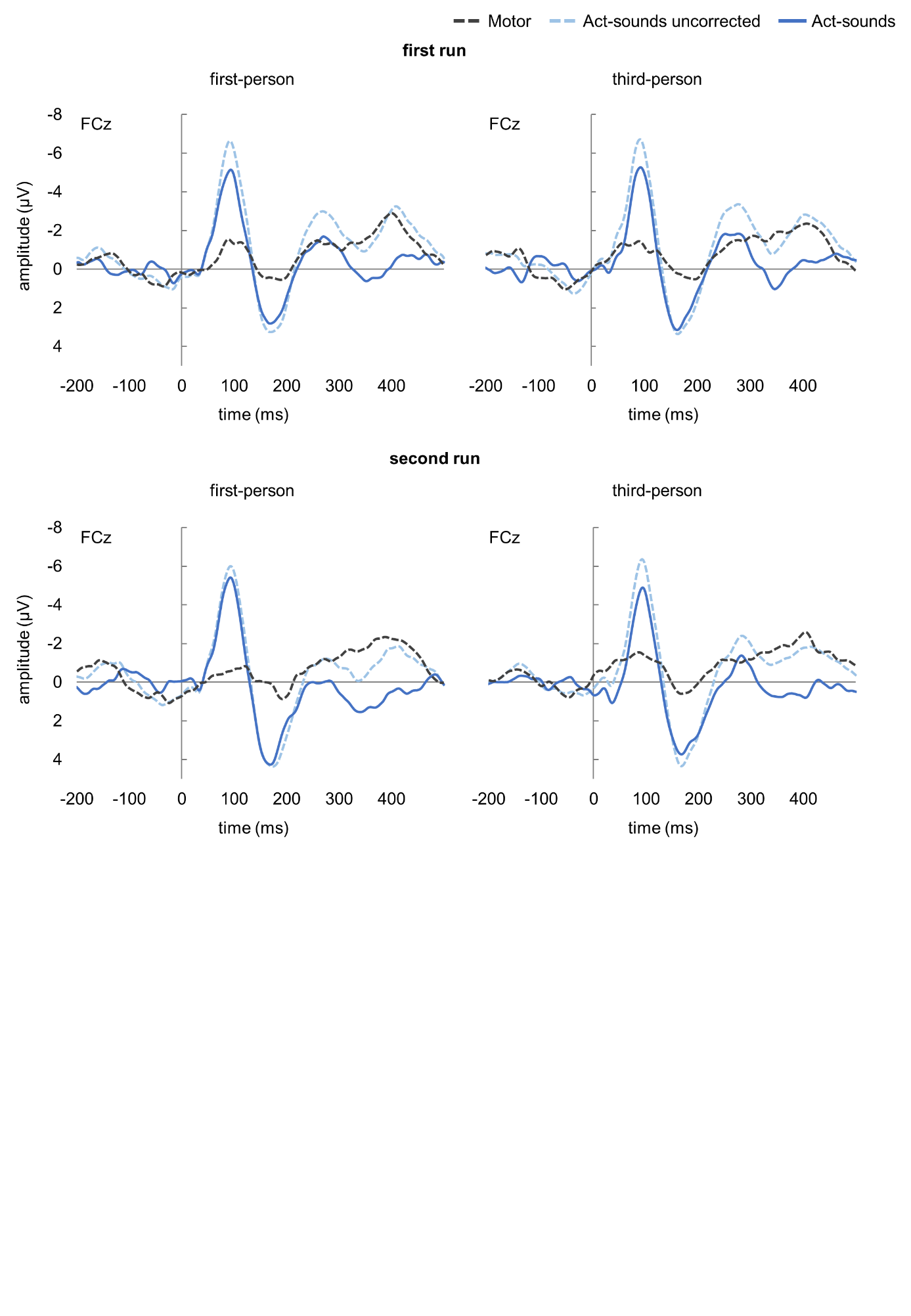


*Supplementary Figure 2.* A. Grand average ERPs for motor-corrected Act-sounds (blue, solid line), motor uncorrected Act-sounds (blue, dashed line) and the Motor-only (black, dashed line) condition, separately for each viewpoint and run.

To explore possible differences of the averaged Motor-only ERPs (see Figure S2) between viewpoints, which were used to correct the signals elicited by Act-sounds for the activity of the movement observation per se, we provide an analysis of mean amplitudes from this condition. Because visual inspection suggested a possible difference between viewpoint conditions in the time frame from 0-99 ms, mean amplitudes were calculated in the averaged signal of each participant (separately for each Viewpoint and Run condition) in ten consecutive 10 ms time windows between 0 ms and 99 ms. Amplitudes were captured at electrodes Fz, FCz and Cz as in the main analysis, and underwent the same outlier rejection procedure. All amplitudes were fitted to the same linear mixed effects model:

$$Amplitude \sim Viewpoint*Run+\left( 1+Viewpoint*Run\left| \mathrm{Participant} \right. \right)+\left( 1 \left| \mathrm{Electrode} \right. \right)$$

The α level was Bonferroni-corrected (.005). The random effect for electrode had to be removed for the first time window (0-9 ms) to avoid a singular fit.

| Mean Amplitude Time Window | *F*(1, 26) | *b* |
| --- | --- | --- |
| 0-9 ms | -3.29* | -0.64 |
| 10-19 ms | -3.78** | -0.74 |
| 20-29 ms | -2.80 | -0.69 |
| 30-39 ms | -2.97 | -0.78 |
| 40-49 ms | -3.50* | -1.01 |
| 50-59 ms | -3.18* | -0.97 |
| 60-69 ms | -2.62 | -0.84 |
| 70-79 ms | -1.88 | -0.66 |
| 80-89 ms | -1.21 | -0.48 |
| 90-99 ms | -0.96 | -0.38 |

Note: Viewpoint main effect statistics.

*p < .005, **p < .001.

Results for the Viewpoint main effect (see table above) showed a significant difference between viewpoint conditions for the time windows 0-9 ms, 10-19 ms, 40-49 ms, and 50-59 ms, but not for the remaining time windows. Parameter estimated revealed more negative mean amplitudes for the third-person compared to the first-person perspective in all cases. The Run main effect (all *p*s > .086) and the Viewpoint by Run interaction (all *p*s > .115) did not reach significance in any time window.

To provide an overview of further potential differences between viewpoints in a larger time range of the signal elicited in the motor-only condition, a similar approach was used to analyze mean amplitudes in ten 50 ms time windows from 0 – 499 ms. This revealed a significant main effect of Viewpoint for the first time window (0-49 ms), F(1, 26) = 14.17, p < .001, b = -0.77, confirming the results of the previous analysis. No other significant effects were found for Viewpoint (all *p*s > .050), Run (all *p*s > .259), or the Viewpoint by Run interaction (all *p*s > .132).

**S3. Grand averaged ERPs to visualize time course effects**

**
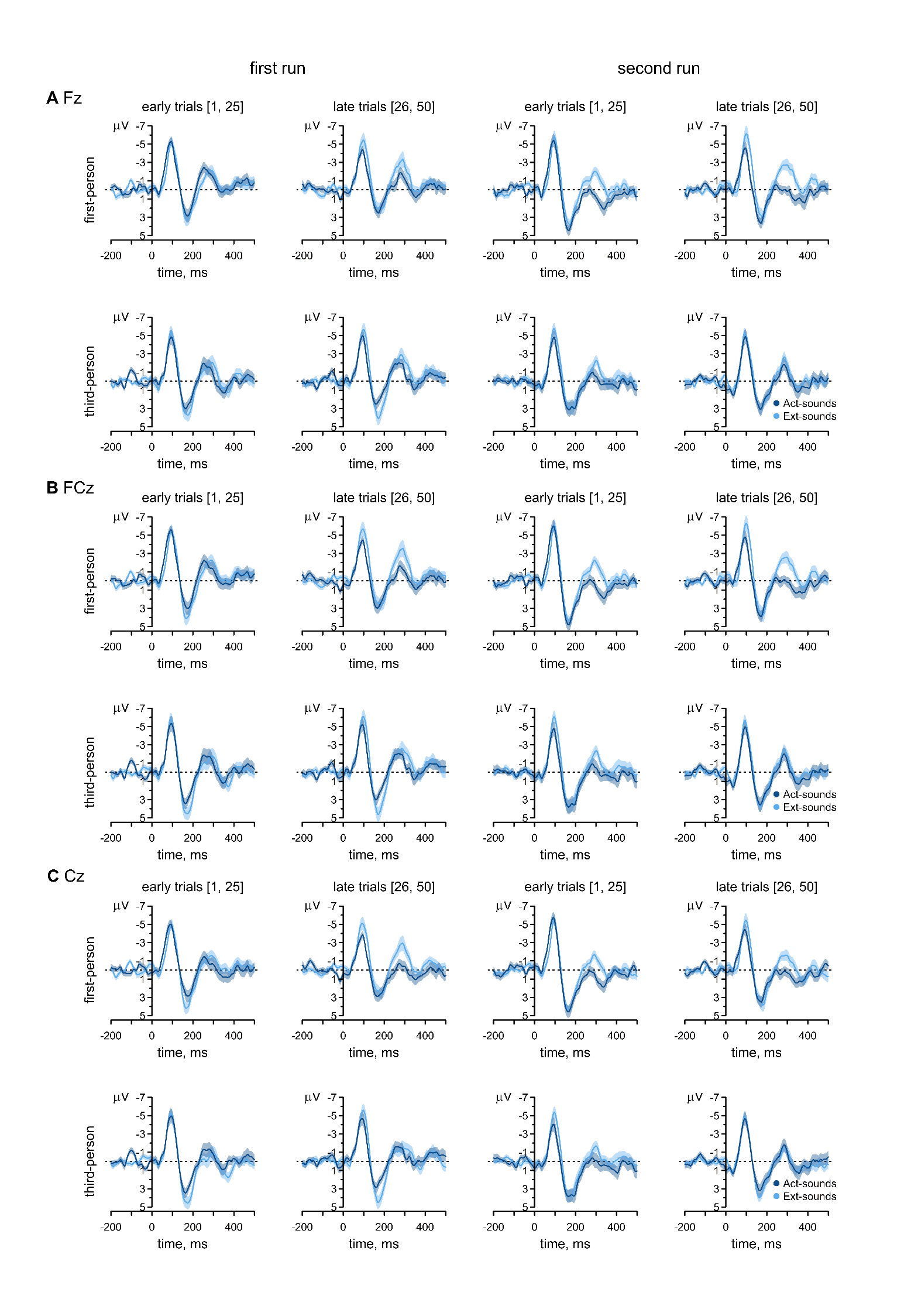
**

*Supplementary Figure 3.* Grand-averaged waveforms at electrode Fz (A), FCz (B), and Cz (C) collapsed over all participants (*N* = 27) showing the ERP in two bins of trials (i.e. early (trial 1 to 25) and late (trial 26 to 50)) of each run as a function of Sound Type (Act-sounds = dark blue, Ext-sounds = light blue) and separately for the first-person (each upper row) and third-person perspective (each lower row) to visualize the time-variant effect pattern. Shaded areas represent one standard error above and below the mean.
